# Implementing dynamic distance-time conversion factors through real-time traffic data for enhancing urban mobility and service accessibility in South Korea

**DOI:** 10.1371/journal.pone.0330266

**Published:** 2025-09-04

**Authors:** Solhee Kim, Taegon Kim, Jeongbae Jeon

**Affiliations:** 1 Department of Smart Farm, College of Agriculture & Life Sciences, Jeonbuk National University, Jeonju, Jeonbuk State, South Korea; 2 Institute of Agricultural Science & Technology, Jeonbuk National University, Jeonju, Jeonbuk State, South Korea; 3 Spatial Information Research Institute, Korea Land and Geospatial Informatix Corporation, Jeonju, Jeonbuk State, South Korea; Novum Research and Innovation Group, LEBANON

## Abstract

This study introduces an expanded methodology for smart regional planning tailored to improve public service accessibility. We develop a city-level distance-time conversion factor (DCF) that utilizes regional characteristics to offer more intuitive estimates of travel times and distances in public service planning. This approach integrates three key variables: road network distances, Euclidean straight-line distances, and minimum travel times derived from both speed limits and actual traffic speeds. The DCF, formulated from the circuity factor (CF) and the delay factor (DF), identifies areas with elevated DCF values, particularly in major metropolitan areas. These metrics serve as critical indicators for densely populated areas, marking a substantial improvement over traditional methods of uniform location planning. Our analysis addresses underdevelopment and population density challenges, underscoring the need for adaptable planning strategies. By incorporating real-time traffic data, the DCF provides insights crucial for strategically developing public infrastructure in high-demand regions. This research enhances the existing smart public service planning frameworks, emphasizing the significance of regional-specific strategies. Ultimately, our findings advocate for a tailored approach to infrastructure development, aiming to create more efficient and responsive public services.

## Introduction

Most East Asian and Eastern European nations face significant demographic challenges characterized by a decline in birth rates and an aging population. The gravity of these changes is escalating, highlighting the need for strategic policy interventions [1–3]. Thus, a paradigm shift is urgently needed to develop responsive measures and proactive adaptation strategies in three key areas: (1) public sector development that supports regional planning, livability, and sustainability [4,5]; (2) changes in land use [6]; and (3) improved commuting patterns [7–9]. South Korea has allocated a substantial financial investment of approximately KRW 380 trillion to address declining birthrates and an aging population. Despite these concerted efforts, South Korea’s population has declined since its zenith in 2020 (51.84 million), marked by a notable inflection point in December 2020 when deaths surpassed births. The Korean Development Institute (KDI) has forecasted a persistent modest population growth rate of −0.1% from 2021 to 2035, followed by an accelerated decline to −1.24% by 2070 [10].

Japan’s demographic landscape has also shown a similar decline, with an average annual birth rate of −0.2% from 2015 to 2020 [11]. China is also expected to see an impending population decline as early as 2027 [12,13], and Europe, where population contraction is projected at an annual rate of −0.3% through 2050 [14]. On a broader scale, the United Nations Population Division (UNPD) has forecasted a global population growth rate nearing 0% by the end of the 21st century. These patterns highlight the urgent need for comprehensive scientific analyses and evidence-based policy frameworks to navigate and mitigate the intricate dynamics of these demographic shifts.

A novel approach to smart regional planning is imperative, necessitating a meticulous examination of the current accessibility landscape of public services and a comprehensive diagnostic evaluation [15]. This strategy is essential to effectively respond and adapt to the challenges posed by underdevelopment and population overcrowding, considering the unique characteristics of each region [16,17]. Given the inherent nature of public services, it is incumbent upon the state to ensure the provision of facilities that are geographically accessible and have the requisite capacity to cater to the needs of the entire population [18]. However, in numerous small- and medium-sized cities and rural areas, depopulation has reached a critical juncture, making it arduous for local residents to sustain a desirable standard of living. This has resulted in regional downsizing and communalization [19].

In contrast to smaller regions, metropolitan and large cities resembling mega cities grapple with population surges and industrial influx from the peripheral areas, contributing to a pronounced polarization [20]. As a result, there is a stark contrast between areas striving to maintain basic community conditions, encompassing education, healthcare, and safety, and areas experiencing overcrowding. Although the provision of fundamental public services remains non-negotiable for the convenience and well-being of local residents, commercial services have dwindled in rural locales. To address these complex dynamics, governments, regional development researchers, and other stakeholders must undertake fundamental research to derive a factor or representative value capable of intuitively diagnosing residents’ access to public services. The insights from research can inform the implementation of efficient and palpable smart planning strategies [21].

Future regional planning must evolve to encompass distance-time accessibility by introducing a temporal dimension to the conventional physical distance measurement. Conventional place-based accessibility studies often assume that the optimal route between two points (Origin-Destination) is the shortest physical distance or travel time [22,23]. While an objective function can minimize travel distance or time, many studies have predominantly adopted the optimization method focused on reducing physical distance. This approach has led to the creation of origin-destination (O-D) matrices based on this metric. To enhance temporal accessibility assessments, recent studies have incorporated attribute information on the legally allowed speed limit of road segments and travel impedance [24]. However, the resulting temporal accessibility approximates the fastest possible travel time within the stipulated speed range [25–27]. However, discrepancies exist between the actual travel times based on the legal speed limit and real-time traffic conditions of the road network. Thus, to evaluate actual accessibility based on travel time, the road network map should integrate information on the real-time travel speed of vehicles. Given that real-time travel speed hinges on dynamic factors such as traffic volume, congestion, signal waiting times, and temporal variations, access to real-time traffic flow information is indispensable for accurate assessments [28–31]. Furthermore, recent studies have shown that the expansion of transportation infrastructure can significantly reshape land accessibility and amplify regional development disparities, particularly in rapidly transforming urban regions [32]. Also, Scenario-based approaches that combine spatial accessibility with urban dynamics offer valuable insight into how future planning strategies can be stress-tested against demographic and infrastructural changes [33].

This study aims to establish a city-level conversion factor that facilitates an intuitive estimation of actual travel times and distances, utilizing both the Euclidean distance metric and real-time traffic data. We identify the minimum travel distances, calculated using the road network map and the direct Euclidean straight-line distance to public service facilities. The study then evaluates the minimum travel times by incorporating designated speed limits and observed real-time speeds. The circuity factor (CF) is derived from these calculations, which measure the ratio of the actual travel route to the shortest possible distance. Concurrently, the delay factor (DF) is assessed by comparing the actual travel times against those anticipated under normal speed limit conditions. This research culminates in creating a distance-time conversion factor (DCF), which integrates the CF and DF. This factor is essential for strategic planning and offers critical insights into the accessibility of public services. The approach outlined here provides a solid foundation for enhancing thoughtful regional planning and improving the accessibility of future public services, with a particular focus on the application within South Korea.

## Materials and methods

### Data collection and preparation

**Fig 1:**
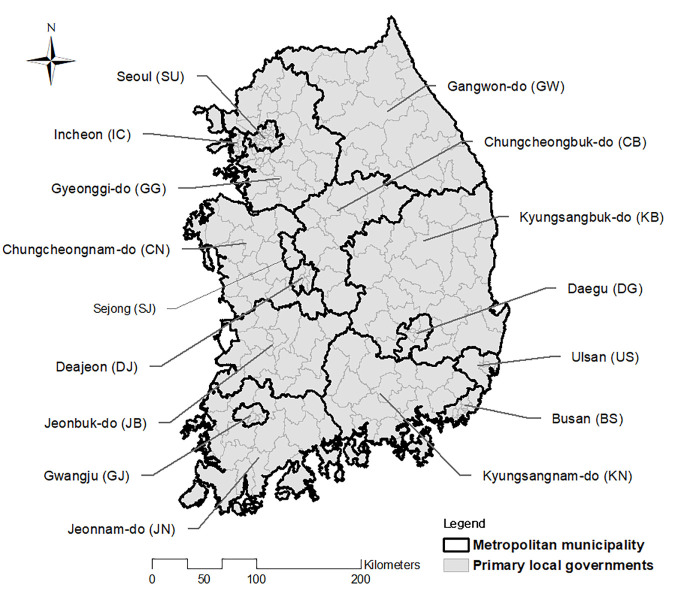
Administrative structure of the study area in South Korea. Map of South Korea showing the metropolitan municipalities (thick black boundaries) and primary local governments (thin gray boundaries), which are the units of analysis in this study. Region names and standard abbreviations (e.g., Seoul [SU], Busan [BS]) are annotated for reference. This figure is created using ArcMap (version 10.2) and used publicly available vector shapefiles for Korean administrative divisions, downloaded from the National Spatial Data Infrastructure Portal (http://www.nsdi.go.kr).

This study focuses on the accessibility of public services in South Korea to evaluate distance-time conversion factors considering various socio-traffic data. The socio-data represent the population and public service facilities, and the traffic data consists of both the road network map and real-time traffic flow data. These necessary data should be available at least at the city level (operating at the primary local government level) under the metropolitan municipality (hereafter, the county) (see Fig 1).

Population data were obtained from the population grid provided by the National Geographic Information Institute (NGII), which provides information on people living within a grid of 100×100 meters [34]. The data for this study covered 98.9% (469,881 grids) of the 474,904 grids in South Korea, excluding island areas with no vehicle access.

Data used for analysis in this study consisted of facility information for public services, including administration, education, finance, healthcare, and safety facilities (Table 1). The address of each facility was collected and converted to the transverse mercator (TM) coordinate system of the GRS80 (Geodetic Reference System 1980) ellipsoid through geocoding [35]. We selected government offices for administrative functions and post offices for logistics functions as representative facilities of each basic local government service. The Ministry of the Interior and Safety (MIS) provides address information for government offices [36], and the Korea Post (KP) provides address information for post offices. We also selected elementary, middle, and high schools as representative facilities for education, with address data provided by the Ministry of Education (ME) [37]. For safety, we selected 119 safety centers and police stations, with address data provided by the National Fire Agency (NFA) [38] and the Korean National Police Agency (KNPA) [39], respectively. For healthcare, we selected hospitals as representative facilities and used data from the National Medical Center (NMC) [40]. For finance, we selected banks as representative facilities and used data on financial institution locations provided by the Open Data Portal (ODP) as an open application programming interface (open API).

**Table 1:** Summary of datasets used for accessibility analysis, including public service facilities, population distribution, and traffic flow.

Category		Spatial Resolution	Number of data		Temporal Resoulation	Source
			Records	Missing/ Invalid		
**Public Facility data**						
Education	Elementary school	Address (Point)	6,041	221	Static	ME
	Middle school	Address (Point)	3,156	83	Static	
	High school	Address (Point)	2,307	46	Static	
Safety	119 safety center	Address (Point)	1,762	59	Static	NFA
	Police station	Address (Point)	703	34	Static	NPA
Administration	Post office	Address (Point)	2,545	84	Static	KP
	Government office	Address (Point)	271	1	Static	MIS
Healthcare	Hospital	Coordinate (Point)	1,456	10	Static	NMC
Finance	Bank	Coordinate (Point)	7,184	95	Static	ODP
**Population data**						
Population		Raster Grid (100 × 100 m)	469,881	5,023	2020	NGII
**Traffic flow data**						
Road network map		Polyline (Link ID, Link segment)	531,843	–	Static	ITS Korea
Traffic speed		API (Text, Link segment)	358,143	–	5-min interval (aggregated hourly)	MOLIT

All addresses were geocoded and reprojected to the national standard coordinate system (GRS80-TM). Traffic speed records were collected at 5-minute intervals and smoothed by hourly averaging. ODP and TRIS datasets were accessed through open APIs.

All data sources and formats are described in Table 1 and are accessible via the repository listed in the Data Availability Statement.

Traffic information included road network maps and real-time traffic speed data to evaluate accessibility based on the traffic network. Collecting real-time speed information on roads allowed for accurate temporal distance calculations. In South Korea, road network maps are provided as standard node links in the Intelligent Transport System in Korea (ITS Korea) [41]. Real-time traffic speed data are available at the Traffic and Road Information Service (TRIS), operated by the Ministry of Land, Infrastructure, and Transport (MOLIT) [42]. The TRIS provides real-time information about traffic flows, construction, accidents, and other unexpected events as an open API interface. Traffic flow information automatically measures the passing speed of vehicles using cameras installed on roads to collect traffic flows and provide information in connection with the road network by geo-localizing the data. This traffic flow information includes approximately 3–4 gigabytes (GB) of daily traffic data recorded at 5-minute intervals. Each record includes the date, time, link ID, and instantaneous speed. The spatial resolution of the data corresponds to road segments (links) defined in the national road network standard node-link database. To ensure computational feasibility and enhance temporal stability, the raw 5-minute data were aggregated to hourly averages. Outlier speeds, exceeding ±2 standard deviations from the median at each time point, were filtered to smooth extreme fluctuations, using a robust z-score method. The cleaned and aggregated data were then joined to the road network using the link ID for further accessibility analysis

### Data analysis

Distance is an essential factor for accessibility analysis. It is usually a numerical representation of the distance between two points, and can be defined as the physical distance or an interval of time [43,44]. The most common distance estimation method is the Euclidean distance measure, which uses the Pythagorean theorem to determine the distance between two points on a coordinate plane using analytic geometry. This method has the advantage of being computationally simple compared to other methods [45]. However, Euclidean distance is calculated independently of the configuration of the road network installed based on terrain conditions such as mountain ranges, lakes, and rivers, which is different from the actual physical distance.

To address these issues, studies have estimated the exact actual road distance by multiplying the Euclidean distance by a certain factor [46–49]. One of the factors is the circuity factor (CF) from point i to point j (${CF}_{ij}$), defined as the ratio of Euclidean straight-line distance ($e_{ij}$) to the distance along the actual road network ($n_{ijk}$) considering different road types such as highways and urban streets indicating the weight factor for road types for each segment k ($w_{k}$) [45,50–52]. However, the recent development of geographic information systems and big data has made it possible to accumulate large amounts of data and more accurately estimate the factors used in distance estimation and introduce the concept of time [52–54].


$$\begin{array}{c} {CF}_{ij}=\frac{\sum \left(w_{k}\cdot n_{ijk}\right)}{e_{ij}} \end{array}$$ (1)

**Fig 2:**
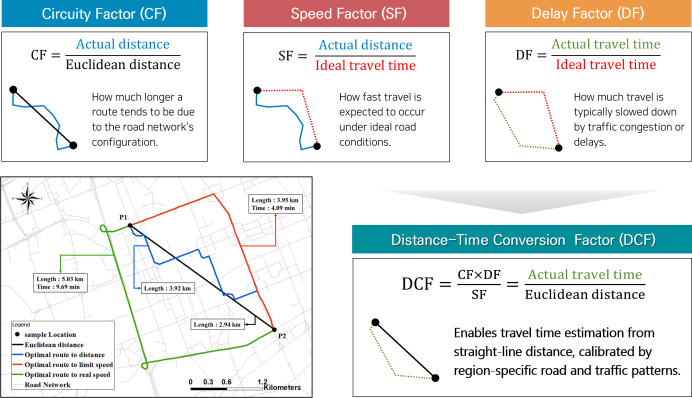
Optimal routing and conceptual components of the distance-time conversion factor (DCF): circuity, speed, and delay. Optimal routes between two locations (P1 and P2) based on different objective functions: shortest Euclidean distance, shortest network distance, and minimum travel time under both legal speed limits and real-time traffic conditions. Each component of the DCF—Circuity Factor (CF), Speed Factor (SF), and Delay Factor (DF)—is visually represented through example paths. The bottom map shows how these routes differ depending on whether the objective is to minimize physical or temporal travel cost. The final DCF is computed by integrating these three components.

We analyzed accessibility from the population grid to the nearest public facilities using network analysis based on the road network. We used the network analyst extension in geographic information systems (GIS) in ArcGIS^TM^, which is an advanced vehicle routing tool to maximize transportation efficiency. We analyzed the accessibility of the nearest public facilities based on the road network following Jeon, Kim *et al.* [55]. We used an objective function to calculate the CF that minimizes the travel distance according to the road network map. However, in terms of time, the analysis should be performed with an objective function that minimizes travel time, and the results vary as shown in Fig 2.

Similar to CF, the delay factor (DF) is defined as the ratio of the actual travel time to the ideal travel time along a road segment $n_{ij}$ (Eq. (2)). The ideal travel time is calculated based on the speed limit, assuming no delays. The actual travel time (${TA}_{n_{ij}}$) is the observed actual travel time, which can vary depending on the time of day. Also, the ideal travel time (${TI}_{n_{ij}}$) is the theoretical ideal travel time, assuming no traffic congestion, based on the speed limits.


$$\begin{array}{c} {DF}_{ij}=\frac{{TA}_{n_{ij}}}{{TI}_{n_{ij}}} \end{array}$$ (2)

The ${TA}_{n_{ij}}$ typically varies at different times of the day due to changes in vehicle density and traffic patterns. Specifically, segments are analyzed during rush hours in the morning (7:00–8:59) and evening (17:00–19:59), as well as during less congested periods in the late morning (9:00–11:59), afternoon (12:00–16:59), and night (21:00–22:59). The actual travel times, ${TA}_{n_{ij}}$, are expected to increase during rush hours due to higher vehicle densities, affecting the ${DF}_{ij}\;$ alues. Conversely, during off-peak hours, ${TA}_{n_{ij}}$ approaches ${TI}_{n_{ij}}$, indicating a more efficient flow of traffic.

Speed should be traditionally represented as distance traveled divided by the time taken to travel that distance at the speed limit. Thus, a more intuitive representation for the speed factor (SF), which aligns with standard speed calculations, would be shown as Eq. (3). The SF represents the ideal speed at which the distance from point i to point j along the road network can be traversed, assuming travel occurs at the speed limit. It is calculated as the ratio of the minimum road network distance $n_{ij}$ to the ideal travel time ${TI}_{n_{ij}}$, calculated based on the speed limit.


$$\begin{array}{c} {SF}_{ij}=\frac{n_{ij}}{{TI}_{n_{ij}}} \end{array}$$ (3)

Knowing the CF, DF, and SF for each region in advance allowed us to easily estimate the time required to travel during each time period using only the coordinates of two points. The Time-Distance Conversion Factor (DCF) integrates geographic and temporal data to estimate the travel time between two points using predefined regional metrics (Eq. (4)). It is defined by adjusting the combined effect of road layout and traffic delays (expressed by the product of CF and DF) with the average travel speed (SF), providing a practical estimate of travel time.


$$\begin{array}{c} {DCF}_{ij}=\frac{{CF}_{ij}\cdot {DF}_{ij}}{{SF}_{ij}} \end{array}$$ (4)

The DCF means that by multiplying CF and DF, we assess the compounded impact of both road layout inefficiency and traffic delays on travel and then adjust this product by the typical travel speed to convert this impact into a time estimate. By presenting DCF in this manner, it becomes a powerful tool in traffic management and urban planning, synthesizing complex data into actionable insights, which is especially useful for predicting and managing travel times across different regions during various times of day.

## Results

### Characterizing regional accessibility conditions

Our study employed an accessibility analysis framework with the population grid as the origin and each public service facility as the destination. On average, public service facilities are strategically distributed within a 10-kilometer radius of the population grid. This distance can theoretically be covered in approximately 10 minutes based on the road speed limit. However, a notable revelation emerged during our investigation. Based on the actual flow speed of the road, the travel time was approximately 1.5 to 1.6 times longer than the prescribed speed limit. This compelling finding highlights a potential underestimation of the access time when relying solely on the road speed limit for calculations.

Regarding the actual traffic flow rate by time of day, the longest travel time was during the peak hours of 5 pm to 7 pm. The travel time for the morning rush hour period (i.e., going to work) took less time than the travel time during the later evening time (i.e., going home from work). This could be a result of the flexible evening rush hour compared to the more/heavier traffic and slower times. It is also possible that people adjust their departure time between 7 am and 9 am in anticipation of traffic congestion so only a few sections of the road are congested and the travel time is less than that of the evening commute. This result is similar to the study by Jeon *et al.* [55], which showed that the travel time increases continuously from 7 am to 8:30 am, and the peak commuting period is between 8:40 am and 8:50 am, and then decreases again.

Table 2 summarizes the public service facility information and accessibility evaluations including the average accessible distance and traveling time based on speed condition. The most common public facilities in South Korea are banks and elementary schools. We found a significant difference in the accessibility of these two types of facilities. There are about 7,000 banks, but the average distance from the population grid is 9.2 kilometers, which is longer than the distance to other public facilities. The difference may be due to banks having a commercial purpose and being concentrated in densely populated areas. For schools, on average, people can reach one of the 6,000 elementary schools within 3.44 kilometers. This implies that elementary schools are relatively evenly distributed across the country.

**Table 2:** Average accessible distance and traveling time by speed condition for public services.

Facility		Distance (unit: km)		Travel time (unit: min)						
		Euclidian distance	Road distance	Road limit speed	Actual traffic flow speed					
					Rush hour (7–8 am)		Morning (9–11 am)	Afternoon (12−4 pm)	Rush hour (5–7 pm)	Night (9–10 pm)
Education	Elementary school	2.60	3.44	4.01		6.07	6.01	6.16	6.17	6.17
	Middle school	3.38	4.51	5.19		7.87	7.75	8.02	8.04	8.04
	High school	4.40	5.85	6.64		9.88	9.73	10.09	10.14	10.13
Safety	119 safety center	3.43	4.47	5.13		7.97	7.78	8.24	8.27	8.24
	Police station	6.23	8.16	9.09		13.94	13.60	14.50	14.61	14.53
Administration	Post office	2.96	3.80	4.42		6.91	6.76	7.12	7.14	7.13
	Government office	8.12	10.57	11.59		17.73	17.28	18.48	18.57	18.46
Healthcare	Hospital	7.56	9.97	10.86		15.82	15.54	16.24	16.31	16.29
Finance	Bank	7.00	9.20	10.17		14.63	14.44	14.89	14.97	14.98

The distance is divided into Euclidian distance and actual road distance (unit: kilometer). The travel time is indicated by the road speed limit and actual traffic flow speed by period (unit: minute).

### Distance-Time Conversion Factor (DCF) for public service

#### Circuity Factor (CF).

The CF coefficient represents the actual road distance for a traveler to reach a destination based on the actual road network environment compared to a Euclidean straight distance. Ballou et al. (2002) suggested that the global CF is about 1.3 [51]. In South Korea, Kim *et al.* (52) found that the CF is 1.26, calculating the CF with 160 randomly selected points across the country. We analyzed the CF using 466,850 population grids nationwide and found that the CF was 1.309, which is much higher than Kim *et al.* (52) study (Table 3). We expect that this difference is due to the much larger sample size we used to calculate the CF, as well as the more complex road network map compared to Kim *et al.* (52).

**Table 3:** The circuity factor (CF) in South Korea in 2013(Kim et al., 2013) and 2021.

Metropolitan City	Code	Circuity Factor (CF)			Number of data	
		Kim et al. (2013) (A)	This study (B)	Difference (B-A)	Kim et al. (2013)	This study
National Average	KR	1.26	1.309	0.049	160	466,850
Seoul	SU	1.215	1.296	0.081	25	29,681
Busan	BS	1.291	1.310	0.019	16	15,519
Daegu	DG	1.241	1.286	0.045	8	12,791
Incheon	IC	1.269	1.324	0.055	10	14,029
Gwangju	GJ	1.168	1.308	0.140	5	8,450
Daejeon	DJ	1.246	1.311	0.065	5	8,500
Ulsan	US	1.371	1.291	−0.080	5	8,038
Sejong	SJ	–	1.313	1.313	–	3,331
Gyeonggi-do	GG	1.218	1.311	0.093	31	98,222
Gangwon-do	GW	1.435	1.326	−0.109	18	27,319
Chungcheongbuk-do	CB	1.314	1.319	0.005	12	26,287
Chungcheongnam-do	CN	1.275	1.297	0.022	16	41,134
Jeonbuk-do	JB	1.295	1.294	−0.001	14	34,954
Jeonnam-do	JN	1.321	1.318	−0.003	22	43,998
Kyungsangbuk-do	KB	1.298	1.312	0.014	22	52,505
Kyungsangnam-do	KN	1.357	1.337	−0.020	18	45,423

The national average of the CF is the mean value of 16 administrative districts (counties). The number of data indicates the sample size used to analyze the CF of each county.

#### Speed Factor (SF).

We define the speed factor as the speed required to cover the physical distance between two points within the approach time of the speed limit. The national speed coefficient in South Korea is 52.94 (km/hr). Among the 16 counties, Seoul (SE) had the lowest speed factor at 48.68, while Gyeonggi (GG) had the highest at 55.11. Not surprisingly, most metropolitan areas had speed factors that were lower than the national average. Metropolitan cities have a high concentration of people and are likely to experience high traffic volumes. As a result, the speed factor is low due to traffic congestion.

The average SD at the county level in metropolitan cities was a maximum of 59.37 and a minimum of 41.29. The county city (i.e., county seat) with the highest SF was Pocheon, located in the northern part of Gyeonggi-do (GG), and the lowest SF was Yeongdo-gu, located in Busan (BS). For Pocheon, GG is a small urban area with a population of about 150,000 people and is a secondary hinterland adjacent to Seoul (SE) where small- and medium-sized industrial complexes are located. In 2017, a highway opened up from Pocheon to Sejong City (SJ, the administrative capital), and a part of the second ring highway opened. These new routes have dramatically improved transportation to the larger metropolitan areas. As a result, the speed coefficient for these roads is high. Another area, Yeongdo-gu in BS, is an island with 110 thousand residents and is connected to the inland by four bridges. BS is the second largest city in South Korea and has five subway lines. However, there is no subway on Yeongdogu Island in BS. The speed coefficient is higher than other local governments because high traffic volume is moving inland from the island and fewer transportation options are available.

#### Delay Factor (DF).

**Fig 3:**
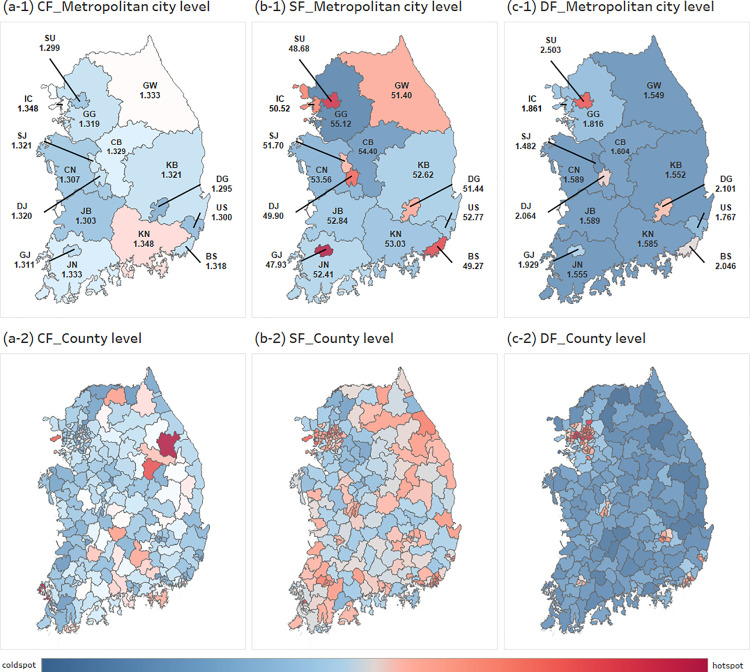
Spatial distribution of circuity factor (CF), speed factor (SF), and delay factor (DF) across metropolitan and county-level regions in South Korea. Red areas indicate relatively higher values, while blue areas indicate lower values, based on the national average. (a) CF reflects road detour levels, (b) SF indicates ideal road speed, and (c) DF captures travel delays caused by traffic congestion. This figure is created using ArcMap (version 10.2). All spatial data were processed and visualized entirely by the authors.

The average DF for South Korea according to the travel time is the highest at 1.694 in the evening (5–7 pm) during the peak rush hour and the lowest at 1.569 in the morning (9–11 am). Of the 16 metropolitan areas, the DF by time of day tends to be highest in 14 areas during the peak evening rush hour for commuting home (5–7 pm) in most areas (Table 4 and Fig 3). Statistics from the Korean Commute Survey show that the average travel time to work is 34.2 minutes, and the average travel time going home is 45.1 minutes. When commute to work, people travel directly from home to work, but when they commute from work, they travel more frequently for personal reasons (e.g., personal meetings, leisure, exercise, and school) and may not go home directly. Thus, DFs during evening commuting hours is higher than during work hours. In addition, due to the recent COVID-19 pandemic and personal quarantine concerns, many people have avoided public transportation, which has likely increased the proportion of lone drivers using private vehicles. This trend may have further increased traffic congestion.

**Table 4:** The delay factor (DF) in South Korea by travel time.

Metropolitan city	Code	Rush hour (7–8 am)	Morning (9–11 am)	Afternoon (12−4 pm)	Rush hour (5–7 pm)	Night (9–10 pm)
National Average	KR	1.612	1.569	1.683	1.694	1.684
Seoul	SU	2.089	1.956	2.386	2.503	2.454
Busan	BS	1.840	1.719	2.034	2.046	2.038
Daegu	DG	1.897	1.815	2.104	2.102	2.077
Incheon	IC	1.752	1.715	1.827	1.863	1.865
Gwangju	GJ	1.756	1.656	1.955	1.930	1.908
Daejeon	DJ	1.727	1.629	1.949	2.066	2.058
Ulsan	US	1.667	1.604	1.749	1.767	1.757
Sejong	SJ	1.425	1.382	1.496	1.482	1.469
Gyeonggi-do	GG	1.692	1.620	1.810	1.816	1.791
Gangwon-do	GW	1.506	1.485	1.543	1.549	1.541
Chungcheongbuk-do	CB	1.545	1.516	1.608	1.604	1.594
Chungcheongnam-do	CN	1.558	1.532	1.587	1.589	1.589
Jeonbuk-do	JB	1.557	1.530	1.588	1.590	1.590
Jeonnam-do	JN	1.529	1.511	1.550	1.555	1.555
Kyungsangbuk-do	KB	1.525	1.504	1.549	1.552	1.553
Kyungsangnam-do	KN	1.542	1.515	1.574	1.585	1.583

Seoul has the highest DF compared to other counties in all time zones, with the highest value of 2.503 during the evening rush hour. Seoul, the capital of South Korea, is a megacity with a population of about 9.55 million people. The high population density causes extreme traffic congestion at all times of the day. In contrast, the region with the lowest DF is Sejong City, with a range of 1.3–1.4. Sejong city (SJ) is an administrative center and a new complex planned city built to mitigate the negative effects of excessive concentration in the capital city of Seoul and to help balance regional development and national competitiveness. Most of the administrative offices located in Seoul were relocated to Sejong City, which was built with careful urban and transportation planning. The result has been a lower DF compared to other local governments. Other large counties with more than 1 million people also tended to have high DF values. Most DFs of the cities in large counties are over 2.00, while the DFs of other counties are in the 1.3–1.9 range. This means that even if the population concentration is not as high as in large counties, the difference between the speed limit and the actual usage speed of the road is about 1.5 times slower.

#### Distance-Time Conversion Factor (DCF).

**Fig 4:**
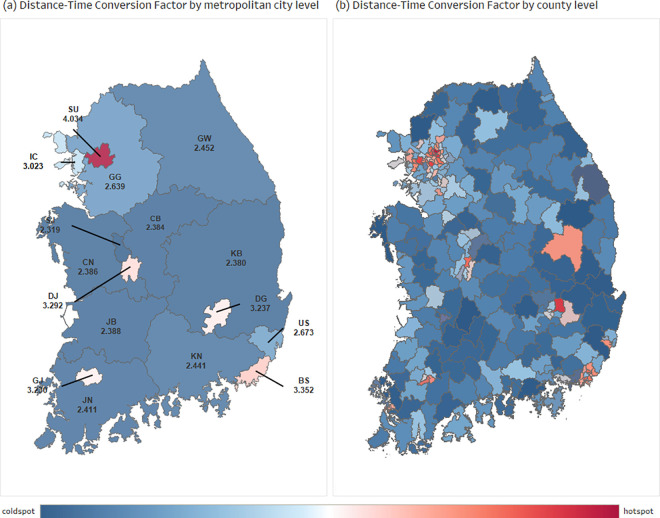
Spatial distribution of the Distance-Time Conversion Factor (DCF) in South Korea. Red areas indicate relatively higher DCF values across (a) the metropolitan city level and (b) the county level, while blue areas indicate lower values, based on the national average. Higher DCF values reflect greater accessibility inefficiency due to longer travel distances or increased delays. This figure is created using ArcMap (version 10.2). All spatial data were processed and visualized entirely by the authors.

The DCF coefficient estimates the travel time based on a straight line between the coordinates of two points. We calculated and visualized the conversion coefficient based on the longest commute time (Fig 4). As with the other coefficients, metropolitan areas have the highest coefficients. Seoul city has the highest DCF, and SJ city has the lowest DCF.

**Fig 5:**
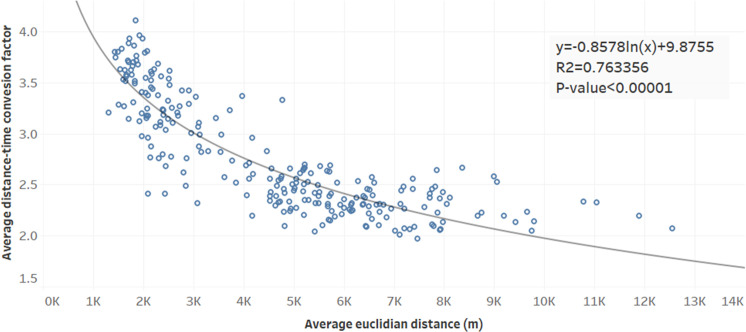
Statistical relationship between average DCF and Euclidean distance across administrative regions. As Euclidean distance increases, the DCF tends to decrease, suggesting that shorter spatial distances are more affected by network circuity and traffic delays, while longer distances exhibit relatively more stable time conversion behavior.

We examined the distribution of DCF and travel distance by administrative region (Fig 5). The results showed that the DCF is higher for short direct routes, while the DCF is lower for long direct routes. Travel time is higher than the straight-line distance due to traffic jams, waiting time at signals, and low-speed traffic. In contrast, fewer factors interfere with traveling to a destination in a long straight line, so the overall impact on time is small.

**Fig 6:**
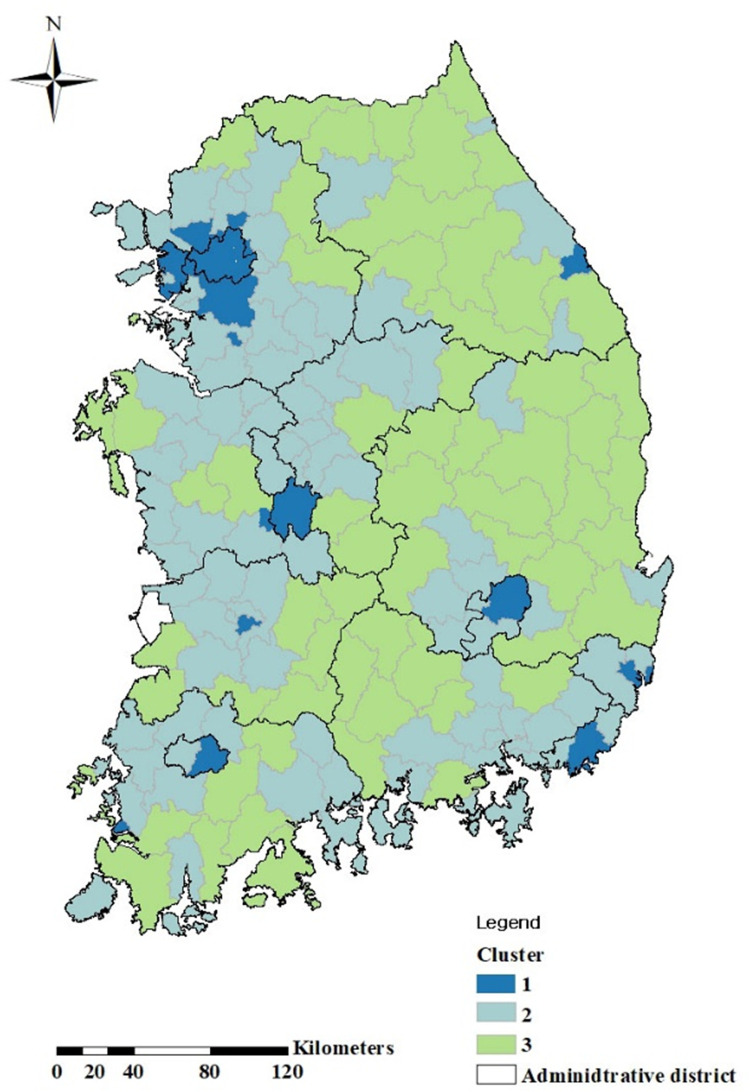
Spatial distribution of regional clusters derived from k-means classification of DCF and accessibility patterns. Cluster 1 (dark blue) includes congested urban areas with high population density and DCF values. Cluster 2 (light blue) covers suburban and mid-sized cities with moderate accessibility gaps. Cluster 3 (green) consists of rural and mountainous regions with long travel distances but low congestion. This clustering supports region-specific infrastructure planning. This figure is created using ArcMap (version 10.2). All spatial data were processed and visualized entirely by the authors.

Based on the distribution of DCF values and straight-line travel distances across administrative regions, we performed k-means clustering to identify distinct spatial patterns in accessibility conditions. The results categorized the regions into three clusters as summarized in Table 5 and visualized in Fig 6. The Cluster 1 includes the five major metropolitan cities including the Seoul (SE)) and Busan (BS), characterized by high population density (277 people on average) and highly concentrated transportation infrastructure, with a road length-to-area ratio of 5.066 km–approximately 4.8 times the national average. These areas exhibit high DCF values primarily due to chronic congestion and delay, and would benefit most from interventions such as traffic demand management, public transit reinforcement, and congestion pricing.

**Table 5:** Attribute information for population, elevation, and road density for three clusters divided by DCF.

Cluster	Attributes of Counties		Population		Elevation (m)	Road density (km/km^2^)
			Total	Average per County		
Cluster 1	Metropolitan	80	25,243,100	277,396	86.525	5.066
	Urban	11				
	Rural	0				
Cluster 2	Metropolitan	0	16,686,543	179,425	145.62	1.917
	Urban	64				
	Rural	30				
Cluster 3	Metropolitan	0	3,185,770	50,567	304.28	0.779
	Urban	11				
	Rural	51				

The areas are divided into three regions by k-means, with Cluster 1 being large cities, Cluster 2 being medium to large cities, and Cluster 3 being small to medium cities.

The Cluster 2 encompasses 74% of urban areas outside the five largest cities, including parts of Gyeonggi Province (GG) and Daejeon (DJ). These areas have moderate population density (average 180 thousand), a road extension ratio of 1.917 km per km^2^, and exhibit moderate DCF values driven by fragmented road networks and uneven facility distribution. Infrastructure investment in connector roads and the redistribution of public services would be most effective in these zones.

The Cluster 3 consists primarily of rural and mountainous regions, such as inland Gyeongbuk (GB) and eastern Gangwon (GW), where the average population is 50 thousand and 63% of the area is classified as rural. These regions also have a higher average altitude, posing physical and financial challenges for road construction. As such, targeted strategies such as strategic road extension, demand-responsive transport, and mobile service delivery are necessary to address accessibility gaps.

This clustering-based typology marks significant progress toward an infrastructure planning approach that reflects the actual operational conditions of regional road networks and traffic dynamics, rather than relying on uniform, distance-based criteria. It enables planners to prioritize interventions not only by absolute travel time but also by structural inefficiencies and temporal instability, supporting equitable and geographically adaptive public service delivery.

## Discussion

This study is in response to the social imbalance of accessibility to public services based on infrastructure planning. The main results showing a discrepancy between the theoretical and actual travel times underscore the need to consider real-world road conditions and flow speeds in accessibility assessments. We applied the concept of speed-distance beyond the dimension of distance attribute information for the accessibility analysis. We also developed an innovative DCF, so it can be applied it to smart planning. This DCF allows us to estimate the actual travel time required to travel the actual road distance even if only the euclidean distance is known between two points.

The CF in our study was about 3.9% higher than Kim *et al.* (52) value, likely due to our use of a more complex road network with spatial disaggregation and a larger sample size concerning pointwise representative public facility locations. Although we used 2918 times more samples than their study, some may consider the difference in CF to be insignificant. However, it is important to note that these results are averaged at the metropolitan city level, and the factors apply to the more spatially disaggregated county level. This approach allows for a more localized and customized design when performing smart planning, especially for policymakers. It provides intuitive numbers to help planners take a more scientific approach to decision-making.

The results also suggest that we should focus more on traffic flow considering the SF, which is based on the legal speed limit of the road and the DF, which is based on the actual traffic flow speed. Planning in densely populated areas using only the speed limit of the road may not reflect the actual road flow. Specifically, the road flow can be underestimated by up to 2.5 times, which can cause many adverse outcomes when making policy decisions. Therefore, we propose that the DF is a useful and essential variable to conduct accurate smart regional policy that reflects the reality of traffic flow.

After clustering the DCF values by administrative region nationwide, a distinct pattern of three clusters emerged. This observation suggests that representative DCF values can be applied systematically to inform public infrastructure planning tailored to specific regions. This marks significant progress towards a public infrastructure plan that authentically mirrors reality, incorporating factors such as the current road network map, road conditions, and actual traffic flow. This approach addresses the limitations of existing uniform location plans.

The integration of Euclidean distance, road network distance, and actual travel time into the DCF framework captures three distinct but complementary dimensions of accessibility. Euclidean distance offers a geometric minimum baseline, serving as a reference to assess spatial inefficiencies. Road network distance reflects infrastructural and topographical constraints by representing the actual connectivity between two points, while actual travel time accounts for temporal variations caused by congestion, signal delays, and traffic dynamics. These dimensions tend to diverge under specific conditions: in complex urban areas, high congestion inflates travel time despite short physical distances; in mountainous or rural regions, road network distance becomes disproportionately longer than Euclidean distance due to limited connectivity. This divergence is empirically demonstrated in Figs 3–6, where urban areas exhibit high DCF values driven by delay factors, and rural areas show high circuity but relatively stable travel times. These patterns support the theoretical rationale for the DCF and affirm that each metric uniquely contributes to a holistic understanding of real-world accessibility.

The selection of specific cities and counties in our analysis was intentional to capture diverse spatial and infrastructural conditions across South Korea. As shown in Fig 3, Seoul (SU) exhibits the highest DF during peak hours, reflecting extreme urban congestion. In contrast, Sejong (SJ) City consistently shows the lowest DF and DCF values in Figs 3 and 4, highlighting the efficiency of planned administrative infrastructure. Yeongdo-gu in Busan, an island district with limited connectivity, displays high circuity but lower speed factors, placing it in Cluster 1 of Fig 6. Meanwhile, Pocheon in Gyeonggi (GG) Province, located in a peri-urban region with recent highway expansion, represents transitional infrastructure conditions, reflected in its positioning in Cluster 2. These regions were chosen to represent a spectrum of accessibility environments; megacities, planned capitals, geographically constrained islands, and suburban hinterlands. This diversity demonstrates the robustness and adaptability of the DCF framework across different regional typologies and supports its potential application in both national and international accessibility studies.

Traditional accessibility assessments often rely on fixed travel-time thresholds (e.g., within 10 or 15 minutes) to define service coverage areas. However, such thresholds implicitly assume uniform travel efficiency across all regions and times, which may lead to overestimated accessibility in congested urban areas or underestimated accessibility in well-connected rural regions. In contrast, the DCF framework accounts for both spatial circuity and temporal delay, offering a more realistic and region-sensitive measure of accessibility. The model is inherently responsive to temporal variations in traffic intensity, as reflected in the variation of DF across different times of day. Although atypical conditions, such as holidays, road closures, or special events, were not explicitly modeled in this study, the DCF approach remains flexible and extensible: adjusting the DF component based on real-time or scenario-based traffic data would enable simulation of such exceptional cases. While this study does not directly compare DCF-based results with conventional fixed-threshold methods, we acknowledge this as a valuable avenue for future research to evaluate the added value and dynamic responsiveness of the DCF model in diverse planning scenarios, including emergency routing and event-specific access planning.

The DCF framework not only addresses critical limitations of conventional accessibility models, such as reliance on posted speed limits, but also holds strong potential for integration into national and urban planning systems. Established speed limits often fail to capture the dynamic nature of road networks, leading to substantial underestimation of access times, particularly in congested or complex traffic environments. This limitation highlights the need for methodologies that incorporate real-time traffic patterns and structural inefficiencies, as achieved by the DCF approach. By quantifying spatial circuity and temporal delay, the DCF model provides a more accurate and region-sensitive measure of accessibility. These advantages position DCF-based indicators as valuable tools in smart city initiatives and infrastructure equity programs. For example, DCF metrics can be embedded into digital twin platforms or urban monitoring dashboards to support real-time accessibility assessments. National initiatives such as South Korea’s Living Area Infrastructure Gap Improvement Program (“Life SOC”) could adopt DCF values as standardized metrics for identifying underserved areas and prioritizing infrastructure investment based on actual service inefficiencies rather than assumed norms.

## Conclusion

In response to the identified shortcomings in current public service accessibility, our study suggests a novel approach: an urban conversion factor designed at the city level utilizing Euclidean distance for the intuitive estimation of travel time and distance. Through a meticulous analysis of road networks, straight-line distances, and real-time travel times, we calculated critical metrics such as the CF and DF, ultimately deriving a comprehensive the DCF for each city. This innovative methodology serves as a foundation for advancing smart regional planning strategies, with the flexibility to accommodate diverse regional characteristics. It effectively addresses challenges associated with underdevelopment and population overcrowding.

The proposed DCF approach is a valuable indicator policymakers and planners can use to evaluate transportation infrastructure relative to population density in urban areas. When transportation infrastructure fails to meet the demands of the population, it results in long travel times and increased costs, which can lead to increased social costs. The findings of this study emphasize that areas characterized by a high DCF are predominantly concentrated in densely populated regions centered around the five largest metropolitan areas, including the capital city. These areas can serve as pivotal indicators for future planning endeavors.

Our study not only highlights the importance of using a city-level conversion factor in addressing public service accessibility issues but also offers a practical and regionally tailored solution to enhance urban planning efficiency. Incorporating real-time travel data and a meticulous analysis of regional characteristics paves the way for a more adaptive and effective approach to smart regional planning.
